# Supplementary material for: The interactome of intact mitochondria by cross-linking mass spectrometry provides evidence for coexisting respiratory supercomplexes
Source: Mol Cell Proteomics. 2017 Dec 8;17(2):216–32. doi: 10.1074/mcp.RA117.000470 (PMC5795388; doi:10.1074/mcp.RA117.000470)
Supplement: Supplemental Data [file supp_RA117.000470_133922_0_supp_23978_fzffwf.zip › spectra_annotation/mito_DR_spectra_annotation/98-1-8-1-12-1.pdf]

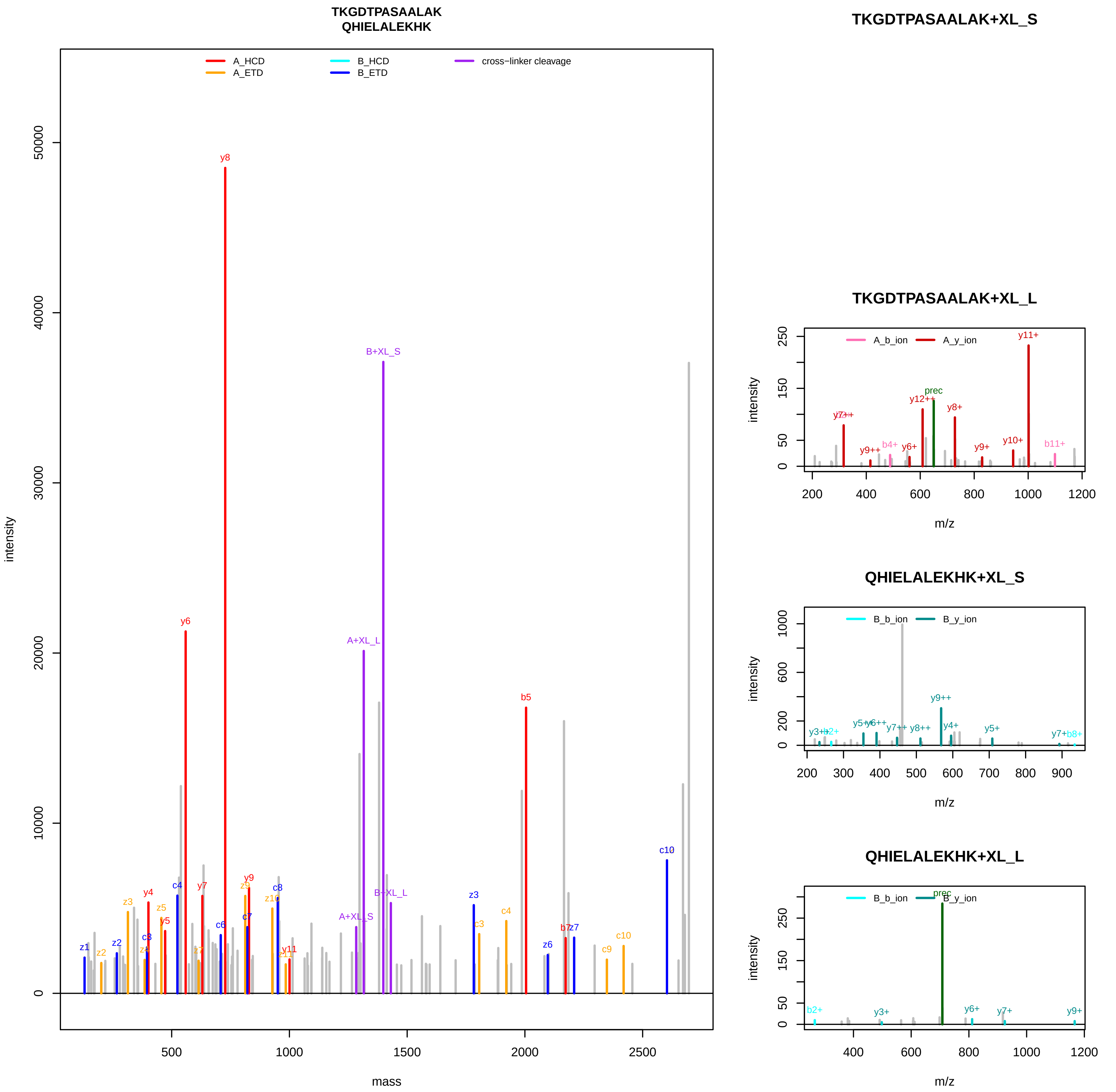
TKGDTPASAALAK
QHIELALEKHK
A_HCD
A_ETD
B_HCD
B_ETD
cross−linker cleavage
intensity
mass
0
10000
20000
30000
40000
50000
500
1000
1500
2000
2500
y8
B+XL_S
y6
A+XL_L
b5
c10
z1
z2
z3
c3
z4
y4
z5
y5
c4
y7
z7
c6
c7
z9
y9
z10
c8
z11
y11
A+XL_S
B+XL_L
z3
c3
c4
z6
b7
z7
c9
c10
TKGDTPASAALAK+XL_S
TKGDTPASAALAK+XL_L
A_b_ion
A_y_ion
intensity
m/z
y11+
prec
y12++
y7++
y8+
b4+
y6+
y9++
y9+
y10+
b11+
QHIELALEKHK+XL_S
B_b_ion
B_y_ion
y9++
y3++
b2+
y5+
y6++
y7++
y8++
y4+
y5+
y7+
b8+
QHIELALEKHK+XL_L
prec
b2+
y3+
y6+
y7+
y9+
